# Limitations of Correlation Coefficients in Research on Functional Connectomes and Psychological Processes

**DOI:** 10.1002/hbm.70287

**Published:** 2025-07-10

**Authors:** Haojie Fu, Shuang Tang, Xudong Zhao

**Affiliations:** ^1^ Shanghai Research Institute for Intelligent Autonomous Systems Tongji University Shanghai China; ^2^ Shanghai Institute of Intelligent Science and Technology Tongji University Shanghai China; ^3^ Faculty of Psychology Southwest University Chongqing China; ^4^ Department of Psychosomatic Medicine, Shanghai East Hospital, School of Medicine Tongji University Shanghai China; ^5^ Clinical Research Center for Mental Disorders, Shanghai Pudong New Area Mental Health Center, School of Medicine Tongji University Shanghai China

**Keywords:** connectome‐based predictive modeling, feature selection, model performance evaluation, Pearson correlation coefficient

## Abstract

In neuroscience and psychology research, the Pearson correlation coefficient is widely used for feature selection and model performance evaluation, particularly in studies examining relationships between brain activity and psychological behavior indices. However, when predicting psychological processes using connectome models, the Pearson correlation has three main limitations: (1) it struggles to capture the complexity of brain network connections; (2) it inadequately reflects model errors, especially in the presence of systematic biases or nonlinear error; and (3) it lacks comparability across datasets, with high sensitivity to data variability and outliers, potentially distorting model evaluation results. To better assess model performance, it is crucial to combine multiple evaluation metrics, such as mean absolute error (MAE) and root mean square error (MSE), which capture different aspects of model quality. Additionally, baseline comparisons, such as using the mean value or a simple linear regression (LR) model, provide an essential reference for evaluating the added value of more complex models. This approach offers a more robust and comprehensive analysis of functional connectomes and psychological processes.

## Graphical abstract

This study highlights the limitations of the correlation coefficient in functional connectome and psychological process modeling, emphasizing its inability to capture nonlinear relationships, address systematic biases, and ensure comparability. By proposing comprehensive evaluation metrics and baseline comparisons, it offers practical guidance for enhancing the reliability of future model assessments.

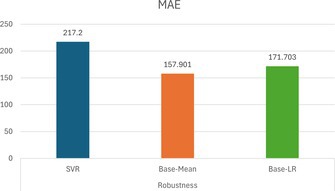

## 1 Introduction

Understanding the relationship between brain functional connectivity and psychological processes is crucial for uncovering the mechanisms underlying human behavior and mental functions in neuroscience and psychology research. Connectome‐based predictive modeling (CPM), as a key method, enables researchers to construct models that explain and predict psychological processes by analyzing relationships between brain imaging data and behavioral or psychological metrics (Ovando‐Tellez et al. 2022; Shen et al. 2017; Zhang et al. 2023). However, the effectiveness and reliability of these models depend on appropriate evaluation methods, which directly impact the scientific value and interpretability of research findings.

The typical CPM workflow consists of several sequential steps: (i) feature selection, used to identify brain functional connections relevant to the target psychological process; (ii) feature summarization, which integrates the selected connectivity features; (iii) model building, where a predictive model is constructed based on the integrated features; and (iv) assessment of prediction significance, to evaluate the model's predictive performance. In these models, the Pearson correlation coefficient is widely used for feature selection and model performance evaluation, particularly in studies examining the relationship between brain activity and psychological behavior indices (Amos et al. 2024; Zhang et al. 2023). However, the CPM framework generally relies on linear assumptions, limiting its ability to capture complex, nonlinear relationships between brain connectivity and psychological behavior (Shen et al. 2017).

The Pearson correlation coefficient (*r*) is a statistical measure that quantifies the linear relationship between two variables, calculated as the covariance of variables divided by the product of their standard deviations. This coefficient ranges from −1 to +1, indicating the strength and direction of association between variables (Pearson 1897). In neuroimaging research, Pearson's *r* has been widely employed to define functional connectivity by measuring BOLD signals between brain regions (Smith et al. 2013; van den Heuvel and Hulshoff Pol 2010), as well as to evaluate model performance (Rosenberg et al. 2016; Shen et al. 2017; Smith et al. 2013; van den Heuvel and Hulshoff Pol 2010). Our study specifically examines the limitations of the Pearson correlation coefficient when utilized for feature selection and model evaluation in predictive modeling, which should be carefully distinguished from its application in constructing functional brain networks. This distinction is crucial for the accurate interpretation of neuroimaging findings and the appropriate methodological implementation in brain–behavior investigations.

In the stages of feature selection and model evaluation, the Pearson correlation coefficient *r* is widely used to measure the relationship between brain activity and psychological behavior indicators. However, as mentioned earlier, relying solely on the correlation coefficient presents three major limitations: (1) In feature extraction, the Pearson correlation coefficient *r* struggles to capture the complex, nonlinear relationships between brain functional connectivity and psychological behavior (Stephan et al. 2008), thereby limiting its ability to effectively represent the underlying connections; (2) it is insufficient for reflecting model error, particularly in cases of systematic bias or nonlinear error within the model; (3) it lacks comparability across different datasets or studies and is highly sensitive to data variability, making it susceptible to distortion by outliers (Armstrong 2019). These limitations can lead to skewed model evaluation results, ultimately affecting the credibility and practical value of research findings. For example, Ripke et al. (Ripke et al. 2013) found 22 genome‐wide significant genetic variants associated with schizophrenia, yet even the strongest association proved useless as a standalone predictor of the disorder (Poldrack et al. 2020). Therefore, correlation metrics should not be misapplied in predictive evaluation, or solely relied upon for assessing prediction performance (Poldrack et al. 2020).

To overcome these limitations, it is essential to integrate a range of complementary metrics when evaluating model performance. Measures such as mean absolute error (MAE) and mean squared error (MSE) provide deeper insights into the predictive accuracy of models by capturing the error distribution (Gunst and Mason 1977), which cannot be fully captured by the correlation coefficient alone. Additionally, incorporating baseline comparisons—as well as using the mean value or a simple linear regression (LR) model—can help establish a reference point for evaluating the added value of more complex models (Kessler et al. 2016).

A comprehensive literature review conducted in 2022 examined studies published prior to that year and revealed that among 108 studies (Yeung et al. 2022), 81 (75%) utilized Pearson's *r* as their validation metric, while only 16 (14.81%) employed difference metrics. Additionally, 27 studies (25%) incorporated external validation using independent test sets.

To gain further insight into current research practices, we conducted a PubMed search using “connectome‐based predictive modeling” as the key term for publications between 2022 and 2024, aiming to understand the evaluation metrics commonly used in CPM methodology. After excluding irrelevant studies and preprints, a total of 113 articles were included in our analysis. The results are shown in Table 1.

**TABLE 1:** Frequency of evaluation metrics used in CPM studies (2022–2024).

Model evaluation	Frequency (*n*)
Spearman & Kendall	34
Difference metrics	44
External validation	34
Total studies	113

*Note:* Detailed information for each article is provided in Supporting Information 1.

In the period of 2022–2024, 30.09% of articles employed Spearman's correlation or Kendall for calculation. Approximately 38.94% of articles incorporated difference metrics in their evaluation frameworks, while about 30.09% of studies conducted external validation. The use of difference metrics and external validation has seen a slight increase compared to before 2024. However, some articles merely reported difference metrics without detailed discussion, still primarily relying on correlation coefficients as the main criteria for discussion (Lv et al. 2024; Zhang et al. 2023).

This paper aims to address these challenges by critically examining the limitations of Pearson's correlation coefficient in connectome modeling, discussing its impact on the robustness of model evaluation, and proposing an approach that combines multiple performance metrics for a more comprehensive and accurate assessment of brain function–psychological process relationships.

## 2 Exploring and Overcoming Three Key Limitations: Case Studies and Solutions

To verify the limitations of the correlation coefficient in predicting models for functional connectomes and psychological processes, we selected a study utilizing a publicly available resting‐state fMRI dataset (Zhang et al. 2023) as a case example. This study utilized resting‐state fMRI data to analyze the relationship between brain functional connectivity and self‐prioritization, using correlation coefficients and other metrics to evaluate model performance. The shared data and results from this study provide an excellent basis for illustrating the limitations of relying solely on Pearson's correlation coefficient in functional connectome modeling. Through a secondary analysis of the data from this case, we demonstrate several issues associated with relying solely on the Pearson correlation coefficient in functional connectome modeling. Additionally, we show how the introduction of extra error metrics and baseline comparisons can provide a more comprehensive evaluation. The code used in this analysis is available at (https://osf.io/3n5hz/).

### 2.1 Limitation in Capturing Nonlinear Relationships

In the case study, researchers first analyzed whole‐brain functional connectivity to predict variations in self‐prioritization. They applied Pearson correlation to each feature and the self‐prioritization scores within subtraining samples, using a common threshold (*p* < 0.01) to remove noisy edges and retain only those with significant correlations (Zhang et al. 2023). Each edge in the connectivity matrix represents the functional connectivity strength between two brain regions. The resulting correlation coefficients (*r* values) were statistically tested, selecting edges with a significance level of *p* < 0.01—retaining only those functional connections with a significant impact on behavior.

However, both Pearson correlation coefficients and robust regression assume a linear relationship between the independent and dependent variables. Interactions between brain functional networks and psychological processes may involve numerous nonlinear relationships. Previous studies have found that the key features identified by deep learning models differ significantly from those identified by LR models (Sheetal et al. 2020). Thus, relying solely on *r* or other linear‐based metrics for model evaluation may overlook many nonlinear characteristics, failing to capture deeper interconnections between brain regions. This limitation of linear methods can lead to misjudgments in the model's predictive capability, potentially leaving critical neural mechanisms unexplored.

To validate the limitations of the Pearson correlation coefficient, we conducted a secondary analysis using data shared by Zhang et al. (Zhang et al. 2023), attempting to introduce nonlinear feature selection methods into complex brain network connectivity modeling and comparing the results. Models that rely solely on the Pearson correlation coefficient often struggle to capture essential nonlinear connectivity features, thereby limiting their predictive capability. In contrast, incorporating nonlinear correlation coefficients, such as Spearman, Kendall, and Delta, into feature selection can partially address the linear limitations imposed by Pearson's approach. However, it is important to note that these coefficients are not fully capable of capturing all aspects of nonlinear relationships and come with their own specific limitations.

For comparison, we employed multiple correlation coefficients with a selection threshold of *p* < 0.01 to extract features from the entire sample. Subsequently, as in the case study process, we used leave‐one‐out cross‐validation (LOOCV) combined with an internal five‐fold cross‐validation (GridSearchCV) to fine‐tune the SVM model (Prettenhofer et al. 2011). This approach enables us to explore the effectiveness of different correlation coefficients in feature extraction, allowing for a more comprehensive capture of the complexity of brain network connectivity. Additionally, we compared the performance of models using features selected by different correlation coefficients against baseline models, including one that predicted values as the sample mean and a linear regression (LR) model without feature selection, evaluating them in terms of MAE. With future advancements in computational power and the growing use of deep learning, we may consider gradually reducing or even eliminating preliminary feature selection to mitigate the risk of overlooking critical features. The validation results are shown in Table 2. As expected, feature selection using Pearson, Spearman, and Kendall correlation coefficients all demonstrated significant relationships (*p* < 0.001) in terms of *r*. Notably, the SVM‐based models showed substantial improvements when compared to the baseline models, including the mean value prediction and LR model. The best performance was achieved when using Kendall as the feature selection coefficient, with the fewest features selected, yielding an MAE of 39.893. This represented a 28.22% improvement over the mean prediction and a 36.95% improvement over the LR model.

**TABLE 2:** Presentation of model results and performance.

Model example	Correlation method	Number of selected features	*r*	*p*	MAE	Improvement over mean baseline	Improvement over LR baseline
SVM	Pearson	361	0.656	< 0.001	42.662	23.24%	32.58%
	Spearman	300	0.672	< 0.001	40.110	27.83%	36.61%
	Kendall	285	0.677	< 0.001	39.893	28.22%	36.95%

*Note:* Here, *r* represents the Pearson correlation between predicted and true values under LOOCV, while MAE represents the absolute difference between predicted and actual values. “Improvement Over LR” indicates improvement relative to a linear regression (LR) model without feature selection, while “Improvement Over Mean Baseline” compares the model's performance to a simple baseline model that predicts the mean of the target variable.

### 2.2 Limitations in Accurately Reflecting Model Error

In the original case study design, an SVM model with a linear kernel was used to predict psychological processes (Zhang et al. 2023). After training, the study calculated the Pearson correlation between predicted and actual values, mistakenly interpreting it as a definitive measure of model success. The authors concluded that “the stronger the correlation between the predicted and true SPE scores, the more successful the model,” which is a potentially misleading interpretation. This is because Pearson's correlation coefficient only captures the degree of linear association between predicted and actual values, without quantifying the error or the magnitude of discrepancies between them. This limitation is particularly important when assessing the model's generalizability.

Regarding the model's generalizability, the original study validated the model using an elderly population. To compare the model performance, we constructed a mean model and a LR model using the elderly group data shared by the authors (Zhang et al. 2023). The results are shown in Table 3. Table 3 presents a comparison of predictive performance across different models. The baseline model from the original study achieved a significant correlation, with *r* = 0.33 and *p* < 0.01; however, the corresponding MAE was high at 217.2, indicating that despite a significant correlation, the model's prediction error remained substantial. In contrast, the mean prediction model reduced the MAE to 157.901, showing a 37.55% improvement over the baseline. The linear regression model achieved an MAE of 171.703, representing a 26.50% improvement over the baseline, although its correlation was negative (*r* = −0.171) and not statistically significant (*p* = 0.184). These results suggest that relying solely on correlation as an evaluation metric has limitations; even when correlation is significant, the model's predictive accuracy may still be insufficient. In fact, while the model showed promise in terms of correlation, its high MAE indicates that it may not generalize well to other populations or datasets.

**TABLE 3:** Model performance comparison.

Model	*r*	*p*	MAE	Improvement (%)
Reported Baseline	0.33	*P* < 0.01	MAE = 217.2	—
Mean Prediction	—	—	MAE = 157.901	−37.55%
Linear Regression	−0.171	*P* = 0.184	MAE = 171.703	−26.50%

*Note:* “Improvement” represents the percentage decrease in MAE relative to the reported baseline.

#### 2.2.1 Overview of Similar Studies

Many studies on functional connectomes report only the *r* and overlook other performance metrics, which can undermine the validity and robustness of their findings (Feng et al. 2024). Some of these studies mention the limitations of relying solely on correlation in their limitations sections (Yu et al. 2020). Additionally, some researchers have acknowledged the limitations of using correlation alone, and in their work, they combine r with other model performance indicators, such as the Akaike information criterion (AIC) (Jiang et al. 2023). These metrics focus primarily on the model's goodness‐of‐fit and complexity, but they overlook the need to further evaluate the model's predictive accuracy and assess the prediction error. Studies that incorporate MAE (Amos et al. 2024) and MSE (Chen et al. 2024) often discuss error metrics less extensively due to the lack of baseline model comparisons. Additionally, comparing with baseline models is equally important in this regard. The developed model should be compared with baseline models, and the performance should be reported (Azizi et al. 2023).

### 2.3 Limitation in Ensuring Reliable Comparability

**FIGURE 1:**
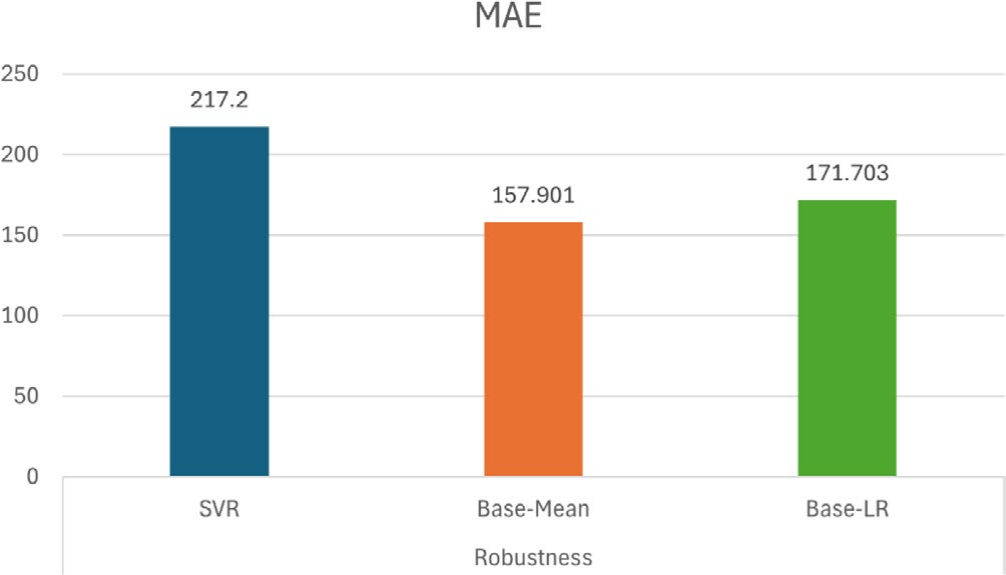
Comparison of model performance: MAE results for case study and baseline models.

The limitations of using the correlation coefficient as an evaluation metric lie in its sensitivity to dataset variability, making it difficult to draw reliable comparisons across different datasets. In addition to this sensitivity issue, it may also be susceptible to systematic biases. Rosenberg recognized this issue and employed robust regression to mitigate the influence of outliers (Rosenberg et al. 2016). However, systematic biases are often overlooked in such evaluations. For instance, in a study on addiction, despite relatively high correlation coefficients, the lack of reported error metrics prevents us from determining whether the results account for systematic biases (Feng et al. 2024). A reliable model should not only achieve statistical significance in the correlation coefficient but also perform better in error metrics compared to baseline models such as mean prediction or LR. Figure 1 presents the MAE results from the generalization validation section of the original case study, compared with the mean model and the LR model. As shown, the case study model performs worse in terms of MAE than both the mean prediction and LR baseline models. Therefore, it can be inferred that the model has significant issues with generalizability. However, the overemphasis on *r* in the study often leads to the neglect of important insights provided by error metrics.

The comparison of model performance should employ a comprehensive evaluation approach, which, in addition to considering the correlation coefficient, also includes error metrics such as MAE and MSE, alongside comparisons with baseline models. This ensures a more holistic assessment of the model's predictive capability and error characteristics (Legates and Davis 1997). These error metrics can reveal how the model performs under different conditions, helping researchers identify potential issues such as systematic biases or overfitting, and ultimately providing more actionable insights for model optimization. When evaluating model performance, multiple metrics are needed (Poldrack et al. 2020).

### 2.4 Cross‐Study Validation: Reanalysis of Connectome‐Based Dishonesty Prediction

To demonstrate the broader applicability of our methodological concerns, we reanalyzed data from an independent connectome study by Speer et al. (Speer et al. 2022), which investigated individual differences in dishonesty using connectome‐based predictive modeling (CPM) on resting‐state functional connectivity. This study provides an ideal validation case as it employed similar methodological approaches but focused on a different behavioral phenotype in a separate cohort. The preprocessed dataset comprised 91 participants with 136 connectivity features. We employed the Lasso regression methodology from the original study while supplementing our analysis with mean and linear regression baseline models and additional evaluation metrics (MAE and RMSE). The results are shown in Table 4.

**TABLE 4:** Performance comparison of different prediction methods.

Method	*r*	*p*	Spearman r	MAE	RMSE
Mean Baseline	—	—	—	16.84	19.35
Linear Regression	0.346	0.189	0.325	18.31	21.67
Lasso	0.404	0.121	0.416	15.99	18.52

*Note:* Given that Lasso regression inherently performs feature selection through L1 regularization (shrinking many coefficients to zero), comparative analyses of different feature selection methods were considered unnecessary and therefore not conducted.

Using 75 samples for training and 16 for testing, we obtained an out‐of‐sample Pearson correlation of *r* = 0.404 (*p* = 0.121), consistent with the original findings. Compared to a mean baseline model (MAE = 16.84, RMSE = 19.35), the Lasso model achieved MAE = 15.99 and RMSE = 18.52, representing a modest 5.0% improvement. When contrasted with a standard linear regression baseline (*r* = 0.346, *p* = 0.189, MAE = 18.31, RMSE = 21.67), the Lasso model demonstrated a 12.7% reduction in prediction error. These results suggest that while the original study reported significant findings through permutation testing (Speer et al. 2022), our evaluation reveals that these improvements are limited in scope. Notably, the relatively small sample size in this study contributes to the nonsignificance of Pearson correlations across many testing conditions.

This validation underscores our central argument that connectome‐based prediction studies require comprehensive evaluation beyond simple correlation reporting, including multiple performance metrics and appropriate baseline comparisons to accurately assess practical predictive value.

## 3 Comprehensive Evaluation Method Suggestions

In climate science, the limitations of using correlation coefficients alone were recognized as early as 1997, with a call for the combined use of *r* with error metrics like MAE and MSE (Legates and Davis 1997). Recent ecological studies have also acknowledged the limitations of relying solely on correlation coefficients (Wadoux and Minasny 2024). Initially, the consistency coefficient was designed to assess the reproducibility of repeated trials for the same variable, not to measure model accuracy. In neuroscience, where sample sizes are often small and the data is more sensitive to outliers, caution is needed to avoid over‐relying on r as an evaluation metric. The consistency prediction model (CPM) has highlighted the limitations of *r* and suggested including metrics like MSE to assess model performance (Ovando‐Tellez et al. 2022). However, many studies still neglect to incorporate error metrics, making it difficult to assess the true quality of their models.

To more comprehensively evaluate model performance in neuroscience and psychology research, we recommend combining the use of the correlation coefficient (*r*) with error metrics that can reveal the model's error patterns, such as whether there are consistent biases or excessive sensitivity to specific inputs (e.g., MAE, MSE). As highlighted in “Ten Simple Rules for Predictive Modeling of Individual Differences in Neuroimaging” (Scheinost et al. 2019), Rule #5A emphasizes that while correlation coefficients describe the strength of linear relationships in model predictions, error metrics provide a better reflection of model bias and accuracy. This combined approach helps mitigate the limitations of relying on a single metric, offering a more comprehensive model evaluation.

In recent years, deep learning techniques have begun to demonstrate unique advantages in neuroimaging analysis (Chen et al. 2023; Wang et al. 2024; Yan et al. 2022), with the significant benefit of automatically capturing complex nonlinear relationships between neural features, while avoiding cumbersome feature preselection steps required in methods such as CPM (Sui et al. 2020). However, these advanced computational methods still face multiple challenges in neuroscience applications: first, the inherent “black box” nature of neural network models severely constrains clear interpretation of neurobiological mechanisms (Schulz et al. 2020); second, the large‐scale datasets typically required for effective training of deep models far exceed the collection capabilities of most existing neuroscience studies (Bruin et al. 2024). Facing these trade‐offs, researchers need to carefully select the most appropriate analytical methods according to specific scientific questions, data characteristics, and interpretation requirements.

## 4 Limitations and Future Directions

Several limitations exist in the current study. First, despite incorporating two case studies, the findings may not fully generalize to all connectome‐based prediction research. The field encompasses diverse methodological approaches, preprocessing pipelines, and behavioral domains, which may exhibit methodological issue patterns different from those observed in this analysis. Second, data accessibility constraints limited the ability to reanalyze a broader range of published studies. Third, the methodological considerations proposed here cannot address all potential methodological challenges in connectome‐based prediction research.

Future work should extend these evaluations to a wider range of connectome studies encompassing different behavioral domains, clinical populations, and analytical methods to further refine best practices in the field. Researchers are encouraged to make their data and code publicly available to facilitate comparative methodological assessments. Such transparency would not only enhance reproducibility but also accelerate methodological improvements across the connectome‐based prediction literature, ultimately strengthening the reliability and validity of brain–behavior relationship findings.

## 5 Conclusion

Through a comprehensive analysis, this paper highlights the limitations of relying solely on Pearson's correlation coefficient *r* as an evaluation metric in the modeling of complex brain network connectivity and psychological processes, particularly its shortcomings in capturing nonlinear relationships, addressing systematic biases, and enabling reliable comparisons across different datasets.

Through case analysis, we propose recommendations for improving research methodologies in neuroscience and psychology, emphasizing the necessity of combining correlation coefficients with error metrics. Additionally, we advocate for the inclusion of baseline model comparisons in all future studies. This comprehensive evaluation approach provides researchers with more holistic feedback on model performance, enhancing the reliability and interpretability of research findings.

## Disclosure

Declaration of Generative AI and AI‐Assisted Technologies in the Writing Process:

During the preparation of this work, the author used ChatGPT (OpenAI) in order to improve the language and readability of the manuscript. After using this tool, the author reviewed and edited the content as needed and takes full responsibility for the content of the publication.

## Ethics Statement

This study utilized publicly available data, and no additional ethical approval was required for this analysis.

## Conflicts of Interest

The authors declare no conflicts of interest.

## Supporting information


Data S1.

## Data Availability

The data for this study were obtained from the article “Decoding individual differences in self‐prioritization from the resting‐state functional connectome” by Zhang et al. 2023, published in NeuroImage (Volume 276, 1 August 2023, 120,205); and the article “Individual differences in (dis)honesty are represented in the brain's functional connectivity at rest” by Speer et al. 2022, published in NeuroImage (Volume 246, 1 February 2022, 118,761). The code used is available at the Open Science Framework (OSF): (https://osf.io/3n5hz/).
